# Chaperone use during intimate examinations in primary care: postal survey of family physicians

**DOI:** 10.1186/1471-2296-6-52

**Published:** 2005-12-21

**Authors:** David H Price, C Shawn Tracy, Ross EG Upshur

**Affiliations:** 1 Primary Care Research Unit, Department of Family and Community Medicine, Sunnybrook and Women's College Health Sciences Centre, 2075 Bayview Avenue, Room E3-49, Toronto, ON M4N 3M5 Canada; 2 Department of Family and Community Medicine, University of Toronto, 263 McCaul Street, 5^th ^Floor, Toronto, ON M5T 1W7 Canada; 3 Department of Public Health Sciences, University of Toronto, 155 College Street, 6^th ^Floor, Toronto, ON M5T 3M7 Canada

## Abstract

**Background:**

Physicians have long been advised to have a third party present during certain parts of a physical examination; however, little is known about the frequency of chaperone use for those specific intimate examinations regularly performed in primary care. We aimed to determine the frequency of chaperone use among family physicians across a variety of intimate physical examinations for both male and female patients, and also to identify the factors associated with chaperone use.

**Methods:**

Questionnaires were mailed to a randomly selected sample of 500 Ontario members of the College of Family Physicians of Canada. Participants were asked about their use of chaperones when performing a variety of intimate examinations, namely female pelvic, breast, and rectal exams and male genital and rectal exams.

**Results:**

276 of 500 were returned (56%), of which 257 were useable. Chaperones were more commonly used with female patients than with males (t = 9.09 [df = 249], p < 0.001), with the female pelvic exam being the most likely of the five exams to be attended by a chaperone (53%). As well, male physicians were more likely to use chaperones for examination of female patients than were female physicians for the examination of male patients. Logistic regression analyses identified two independent factors – sex of physician and availability of a nurse – that were significantly associated with chaperone use. For female pelvic exam, male physicians were significantly more likely to report using a chaperone (adjusted Odds Ratio [OR] 40.62, 95% confidence interval [CI] 16.91–97.52). Likewise, having a nurse available also significantly increased the likelihood of a chaperone being used (adjusted OR 6.92, 95% CI 2.74–17.46). This pattern of results was consistent across the other four exams. Approximately two-thirds of respondents reported using nurses as chaperones, 15% cited the use of other office staff, and 10% relied on the presence of a family member.

**Conclusion:**

Clinical practice concerning the use of chaperones during intimate exams continues to be discordant with the recommendations of medical associations and medico-legal societies. Chaperones are used by only a minority of Ontario family physicians. Chaperone use is higher for examinations of female patients than of male patients and is highest for female pelvic exams. The availability of a nurse in the clinic to act as a chaperone is associated with more frequent use of chaperones.

## Background

Professional guidelines and clinical practice regarding the use of chaperones during intimate physical examinations vary substantially from one jurisdiction to the next. In the United Kingdom, the General Medical Council advises that all patients undergoing intimate exams be offered a chaperone regardless of the sex of the patient or physician [1]. In the United States, on the other hand, there is no clear national standard as each state medical board drafts its own practice recommendations [2,3]. Likewise in Canada, the standards of practice and clinical guidelines vary considerably from province to province. For instance, the guidelines of the College of Physicians and Surgeons of Ontario state that both patient and physician have the right (in non-emergency situations) to insist that a third party be present during intimate examinations, and to insist that the examination be postponed if a third party is unavailable [4].

In recent years, there has been an increasing call by medico-legal societies and medical insurance companies for greater use of chaperones during intimate examinations [5]. Despite this trend, the frequency of chaperone use has generally remained low – although it varies considerably depending on the specific setting and circumstance [6-8]. Another recent trend is that, rather than insisting on the use of chaperones for all intimate examinations, some professional bodies now recommend that the offer of a chaperone be made to patients. This change may reflect the fact that there is great variability in the views of patients toward [9-12], as well as the increased popularity of shared-decision making. There is some recent data indicating an increase in the offering of chaperones [13].

With several notable exceptions [13-15], previous studies of chaperone use in primary care have focused exclusively either on one particular examination such as Pap smears [16] or on one particular patient population such as adolescent females [17] or older women [18]. In a recent editorial, a strong case was made for further study of the use of chaperones during examination of male patients [19].

The objectives of the present study are as follows: to determine the frequency of chaperone use among family physicians across a variety of intimate physical examinations for both male and female patients; to identify the factors associated with chaperone use by family physicians; and to investigate whether these factors vary with the type of examination being performed.

## Methods

### Participants and setting

A stratified random sample, based on gender and geographic location, of 500 family physicians in Ontario, Canada was obtained from The College of Family Physicians of Canada. (All Canadian physicians with certification in family medicine are registered with the College; there are approximately 6,800 registered family physicians in the province of Ontario.) Inclusion criteria specified that participants must be fluent in English and currently practicing family medicine either in an office or walk-in clinic. Family physicians who work primarily in an Emergency Department or as clinical associates in a specialty field were excluded. The study was approved by the Research Ethics Board at Sunnybrook and Women's College Health Sciences Centre, Toronto, Canada.

### Survey instrument and administration

A self-administered questionnaire was developed to collect data on the use of chaperones by family physicians when performing a variety of intimate physical examinations, namely, female pelvic, breast, and rectal, and male genital and rectal. The questionnaire was pilot-tested by family medicine residents and staff physicians at Sunnybrook and Women's College Health Sciences Centre who were asked to comment on the clarity of the questions and to note any information missing from the questionnaire that they felt should be included.

A single mailing consisting of a cover letter and the questionnaire was sent out. Recipients were asked to return the questionnaire in the self-addressed, stamped reply envelope. In order to maintain the absolute confidentiality of the respondents, there were no identifying marks on the surveys.

### Statistical analysis

Data were entered into a spreadsheet for analysis using SPSS for Windows (Version 11.0). With respect to the principal dependent variable of chaperone use, we created a dichotomous "ever/never" variable in which those respondents who reported any use of chaperones in their practice (i.e., always, sometimes, or rarely) are contrasted with those who reported "never" using chaperones in their practice.

A descriptive analysis was undertaken of the physicians' demographic data and their responses to the survey questions. Bivariate analyses were conducted using the chi-squared test and *t*-test as appropriate (a probability level of 0.05 was set for statistical significance). Variables with significant association in the bivariate analysis were included in the logistic regression analysis. We constructed a model for each of the five examinations using both the forward and backward stepwise Wald technique. Both unadjusted and adjusted models were generated. Owing to its *a priori *importance, physician age was forced into every model.

## Results

**Figure 1:**
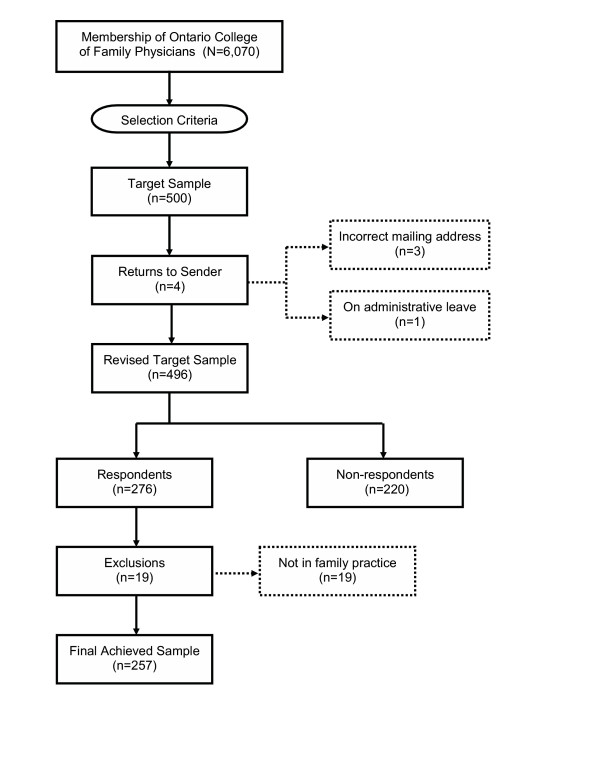
Flow of survey responses.

Of the 500 surveys sent out, four were returned: three owing to incorrect mailing addresses and one because the physician was on administrative leave (see Figure 1). This left a revised target sample of 496. Of these, 279 were returned for a response rate of 56%. Nineteen of the returned surveys were excluded from the analysis because the respondents were not in family practice, thereby yielding a final achieved sample of 257.

The final sample appears to be representative of the target survey population. The age and practice location distributions of our sample closely resembles that of the CFPC National Family Physician Workforce Survey (Janus Survey) [20]. As shown in Table 1, the age distribution of respondents approximates a normal curve. We targeted a 50-50 gender split; however, the achieved sample slightly favours females (54%). The median year of graduation was 1988 and the mean number of years in clinical practice was 14.

**Table 1:** Demographic profile of survey respondents

	**Female**	**Male**	**Total**
**Personal Characteristics**			
Age group:			
<30 years	8 (6)	4 (3)	12 (5)
30–39 years	50 (36)	41 (34)	91 (35)
40–49 years	54 (39)	40 (34)	94 (37)
50+ years	26 (19)	34 (29)	60 (23)
Total	138 (100)	119 (100)	257 (100)
			
Years in clinical practice:			
<5 years	27 (20)	13 (11)	40 (16)
5–14 years	52 (38)	48 (41)	100 (39)
15–24 years	41 (30)	32 (27)	73 (29)
25+ years	16 (12)	25 (21)	41 (16)
Total	136 (100)	118 (100)	254 (100)
			
**Practice Characteristics**			
Number patients seen per week:			
<50	22 (16)	5 (4)	27 (11)
50–99	59 (43)	29 (25)	88 (35)
100–149	33 (24)	47 (40)	80 (32)
150+	22 (16)	36 (31)	58 (23)
Total	136 (99)	117 (100)	253 (101)
			
Practice location:			
Urban	112 (82)	88 (75)	200 (78)
Rural	25 (18)	30 (25)	55 (22)
Total	137 (100)	118 (100)	255 (100)
			
Type of practice:			
Solo	20 (16)	31 (26)	51 (21)
Group	109 (84)	86 (74)	195 (79)
Total	129 (100)	117 (100)	246 (100)
			
Nurse availability:			
Available	87 (64)	86 (73)	173 (68)
Not available	49 (36)	32 (27)	81 (32)
Total	136 (100)	118 (100)	254 (100)

Values are numbers (percentages) of respondents. Totals vary due to missing data and may not sum to 100 due to rounding.

Most respondents worked in a group practice (79%), were remunerated by fee-for-service (88%), and saw between 50 and 150 patients per week (67%). The majority had a nurse available in their clinic to act as a chaperone (68%). Only 6% of respondents had themselves been or knew a colleague who had been the subject of a complaint relating to a physical examination (this small proportion precluded any statistical analysis of the extent to which concerns regarding malpractice are related to chaperone use).

As shown in Table 2, chaperones were more commonly used when examining female patients than male patients. For example, 43% of doctors reported using a chaperone for a female rectal exam compared to only 18% who used a chaperone for a male rectal exam. Overall, regardless of specific exam, use of chaperones was significantly higher for female than male patients, (t = 9.09 [df = 249], p < 0.001). Results also varied between examinations, with a (female) pelvic exam being the most likely (53%) to be attended by a chaperone.

**Table 2:** Frequency of chaperone use

Examination	Percent Reporting Use of Chaperone (n = 257)*
Female Pelvic	53%
Female Rectal	43%
Female Breast	41%
Male Genital	23%
Male Rectal	18%

* For comparison of female examinations versus male examinations, t = 9.09 [df = 249], p < 0.001.

Bivariate analysis identified seven variables (sex, age, availability of a nurse, practice location, practice size, years in practice, and number of patients seen per week) that were significantly associated with the use of chaperones. These variables were entered into the logistic regression models (backward stepwise Wald technique). The results indicated that only two factors – sex of physician and availability of a nurse – were independently associated with the use of chaperones. As presented in Table 3, the odds of a chaperone being used during a female pelvic examination were significantly higher both for male physicians and for those with a nurse available to act as chaperone. The pattern of results for these two factors was consistent across the four other intimate examinations: female rectal (Table 4), female breast (Table 5), male genitalia (Table 6), and male rectal (Table 7). Finally, respondents reported most often using nurses as chaperones (69%), while smaller minorities reported using office staff such as receptionists and secretaries (18%) or family/friends of the patient (10%).

**Table 3:** Use of chaperones during female pelvic exams and logistic regression models

		**Logistic Regression Models**		
		
**Variable**	**% Reporting Use of Chaperone**	**Unadjusted Odds Ratio** **(95% CI)**	Adjusted Odds Ratio* (95% CI)	**P value**
**Sex of physician**				
Female	22.6	1.00^†^	1.00^†^	
Male	87.4	39.29 (16.45 to 93.83)	40.62 (16.91 to 97.52)	<0.001
				
**Age of physician**				
<30 yrs	41.7	1.00^†^	1.00^†^	
30–39 yrs	51.6	0.95 (0.16 to 5.77)	0.57 (0.09 to 3.66)	0.555
40–49 yrs	48.9	0.62 (0.25 to 1.54)	1.17 (0.17 to 8.10)	0.874
50+ yrs	62.7	0.87 (0.34 to 2.20)	1.28 (0.15 to 11.14)	0.821
				
**Nurse availability**				
Not available	30.0	1.00^†^	1.00^†^	
Available	62.4	8.08 (3.24 to 20.16)	6.92 (2.74 to 17.46)	<0.001
				
**Practice location**				
Urban	48.2	1.00^†^	1.00^†^	
Rural	69.1	2.16 (0.87 to 5.35)	2.39 (0.96 to 5.94)	0.062

* Adjusted for physician sex, physician age, nurse availability, practice location, practice size, years in practice, and number of patients seen per week. ^† ^Used as baseline comparison.

**Table 4:** Use of chaperones during female rectal exams and logistic regression models

		**Logistic Regression Models**		
		
**Variable**	**% Reporting Use of Chaperone**	**Unadjusted Odds Ratio** **(95% CI)**	Adjusted Odds Ratio* (95% CI)	**P value**
**Sex of physician**				
Female	16.2	1.00^†^	1.00^†^	
Male	74.1	19.70 (9.58 to 40.51)	19.64 (9.44 to 40.88)	<0.001
				
**Age of physician**				
<30 yrs	25.0	1.00^†^	1.00^†^	
30–39 yrs	41.6	1.73 (0.27 to 11.16)	1.47 (0.22 to 9.82)	0.690
40–49 yrs	45.2	2.91 (0.45 to 18.90)	3.00 (0.45 to 20.03)	0.257
50+ yrs	44.8	1.63 (0.25 to 10.80)	1.49 (0.22 to 10.14)	0.684
				
**Nurse availability**				
Not available	20.5	1.00^†^	1.00^†^	
Available	52.9	6.45 (2.88 to 14.43)	5.67 (2.50 to 12.85)	<0.001
				
**Practice location**				
Urban	37.9	1.00^†^	1.00^†^	
Rural	60.0	1.80 (0.78 to 4.16)	2.09 (0.91 to 4.82)	0.083

* Adjusted for physician sex, physician age, nurse availability, practice location, practice size, years in practice, and number of patients seen per week. ^† ^Used as baseline comparison.

**Table 5:** Use of chaperones during female breast exams and logistic regression models

		**Logistic Regression Models**		
		
**Variable**	**% Reporting Use of Chaperone**	**Unadjusted Odds Ratio** **(95% CI)**	Adjusted Odds Ratio* (95% CI)	**P value**
**Sex of physician**				
Female	15.3	1.00^†^	1.00^†^	
Male	71.4	17.35 (8.66 to 34.75)	17.38 (8.58 to 35.20)	<0.001
				
**Age of physician**				
<30 yrs	33.3	1.00^†^	1.00^†^	
30–39 yrs	38.5	0.69 (0.13 to 3.80)	0.68 ((0.12 to 3.80)	0.660
40–49 yrs	43.6	1.29 (0.24 to 7.08)	1.38 (0.25 to 7.58)	0.711
50+ yrs	44.1	0.73 (0.13 to 4.14)	0.60 (0.10 to 3.43)	0.563
				
**Nurse availability**				
Not available	20.0	1.00^†^	1.00^†^	
Available	50.9	5.93 (2.72 to 12.92)	5.57 (2.55 to 12.17)	<0.001
				
**Practice location**				
Urban	37.7	1.00^†^	1.00^†^	
Rural	54.5	1.32 (0.58 to 2.99)	1.49 (0.64 to 3.46)	0.357

* Adjusted for physician sex, physician age, nurse availability, practice location, practice size, years in practice, and number of patients seen per week. ^† ^Used as baseline comparison.

**Table 6:** Use of chaperones during male genital exams and logistic regression models

		**Logistic Regression Models**		
		
**Variable**	**% Reporting Use of Chaperone**	**Unadjusted Odds Ratio** **(95% CI)**	Adjusted Odds Ratio* (95% CI)	**P value**
**Sex of physician**				
Male	11.8	1.00^†^	1.00^†^	
Female	32.3	3.87 (1.93 to 7.78)	3.87 (1.91 to 7.87)	<0.001
				
**Age of physician**				
<30 yrs	41.7	1.00^†^	1.00^†^	
30–39 yrs	24.7	0.40 (0.10 to 1.53)	0.37 (0.09 to 1.45)	0.153
40–49 yrs	23.9	0.45 (0.12 to 1.72)	0.67 (0.16 to 2.85)	0.585
50+ yrs	13.6	0.20 (0.05 to 0.88)	0.35 (0.06 to 2.03)	0.242
				
**Nurse availability**				
Not available	12.8	1.00^†^	1.00^†^	
Available	26.9	3.49 (1.56 to 7.85)	2.27 (1.03 to 4.98)	<0.05
				
**Practice location**				
Urban	22.6	1.00^†^	1.00^†^	
Rural	23.6	0.88 (0.40 to 1.93)	0.96 (0.42 to 2.21)	0.93

* Adjusted for physician sex, physician age, nurse availability, practice location, practice size, years in practice, and number of patients seen per week. ^† ^Used as baseline comparison.

**Table 7:** Use of chaperones during male rectal exams and logistic regression models

		**Logistic Regression Models**		
		
**Variable**	**% Reporting Use of Chaperone**	**Unadjusted Odds Ratio** **(95% CI)**	Adjusted Odds Ratio* (95% CI)	**P value**
**Sex of physician**				
Male	9.2	1.00^†^	1.00^†^	
Female	25.0	3.56 (1.64 to 7.70)	3.30 (1.53 to 7.08)	<0.01
				
**Age of physician**				
<30 yrs	33.3	1.00^†^	1.00^†^	
30–39 yrs	18.0	0.35 (0.08 to 1.47)	0.32 (0.07 to 1.39)	0.129
40–49 yrs	19.8	0.50 (0.12 to 2.04)	0.71 (0.15 to 3.24)	0.656
50+ yrs	10.2	0.20 (0.04 to 0.98)	0.44 (0.07 to 2.79)	0.382
				
**Nurse availability**				
Not available	7.8	1.00^†^	1.00^†^	
Available	21.6	4.59 (1.75 to 12.09)	3.12 (1.22 to 7.97)	<0.01
				
**Practice location**				
Urban	17.4	1.00^†^	1.00^†^	
Rural	18.5	0.89 (0.37 to 2.12)	0.93 (0.37 to 2.37)	0.876

* Adjusted for physician sex, physician age, nurse availability, practice location, practice size, years in practice, and number of patients seen per week. ^† ^Used as baseline comparison.

## Discussion

The results of this survey indicate that clinical practice concerning the use of chaperones during intimate exams continues to be discordant with the recommendations of many major medical associations and medico-legal societies. Indeed, chaperones are used in only a minority of intimate examinations performed by family physicians in Ontario. Our findings also show significant variance by gender: chaperone use is significantly higher for female patients compared to males. According to these data, the use of chaperones varies greatly by type of examination with the pelvic exam the most likely to be observed by a chaperone. Finally, the availability of a nurse to act as chaperone was strongly associated with chaperone use during intimate examinations.

Our study has several potential limitations. First, physician behaviour was self-reported and therefore may not be a true reflection of actual practice. At the same time, however, our questionnaire was entirely anonymous so there is little cause to believe that respondents did not answer honestly. Second, although a response rate of 56% is now considered high for a physician survey, the possibility of non-response bias does remain. Third, our sample was comprised exclusively of members of the CFPC; consequently, our results may not reflect the practices of general practitioners who have not been certified by the CFPC and who do not participate in ongoing CME as required by the CFPC. We would argue that the similarity of our results to those obtained in other jurisdictions militates against the significance of these latter two potential limitations.

The results of our logistic regression analysis show a strong association between physician sex and the use of chaperones. The adjusted odds ratio for chaperone use during pelvic exams (male physicians 40 times more likely than females) was significantly greater than that reported in a recent US study (males 15 times more likely) [16]. At the same time, however, the overall pattern of findings suggests that the practice of Ontario family physicians regarding chaperone use is similar to that of family physicians in the US [16] and of general practitioners in the UK [15].

Two recent surveys of general practitioners in England indicate that there has been some uptake of the message regarding use of chaperones. Rosenthal and colleagues found that use of chaperones has increased among males physicians over the past two decades, but that use by female practitioners remains low [14]. The study by Conway and Harvey suggests a somewhat different trend with rates of offering chaperones on the increase, but less change in the use of chaperones [13].

Consistent with the finding of the two recent British studies that nurses are the most commonly used chaperones [13,14], our analysis indicates that the availability of a nurse is strongly associated with chaperone use. This finding would appear to have important implications for the current debate regarding primary care reform and the use of a team approach whereby family physicians, nurses, and other professionals work collaboratively as partners in providing care to patients.

## Conclusion

Further research on this issue is needed to understand in greater depth the barriers and facilitators to the use of chaperones during intimate physical exams. It is clear that same sex preference plays a role in the clinician's decision to employ chaperones; however, this may provide a false sense of security, may be inconsistent with best practice recommendations, and may expose clinicians to potential liability. Further investigation is required on the issue of whether malpractice claims and/or concerns function as a determinant of chaperone use [21]. Another valuable complement to the present study would be a survey of patient preferences and experiences as a means to assess the concordance of patient and provider views. And finally, a qualitative analysis of factors promoting and inhibiting the use of chaperones by family physicians would be informative and may prove helpful in developing systems that would ensure the availability of chaperones when needed. Indeed, there is much to be done to close the gap between long-standing medico-legal recommendations and present practice patterns.

## Competing interests

The author(s) declare that they have no competing interests.

## Authors' contributions

DHP initiated the study, participated in the statistical analysis, drafted the first version of the manuscript, and contributed to subsequent revisions. CST co-ordinated the data collection process, participated in the statistical analysis, and contributed to the drafting and revising of the manuscript. REGU conceived the original idea for the study, participated in the statistical analysis, contributed to the drafting and revising of the manuscript, and will act as guarantor. All authors have read and approved the final manuscript.

## Pre-publication history

The pre-publication history for this paper can be accessed here:


